# Reproductive behaviour in free-ranging crested porcupine *Hystrix cristata* L., 1758

**DOI:** 10.1038/s41598-021-99819-3

**Published:** 2021-10-11

**Authors:** Francesca Coppola, Antonio Felicioli

**Affiliations:** Department of Veterinary Sciences, University of Pisa, Viale delle Piagge 2, 56124 Pisa, Italy

**Keywords:** Zoology, Animal behaviour

## Abstract

Previous data on crested porcupine reproduction biology were mostly collected in captivity or semi-captivity due to its elusive, burrowing and mainly nocturnal habits. Between 2017 and 2019 the reproductive behaviour (i.e., intrapair mounting and copulation, birth and parental care) of free-ranging crested porcupine was documented and described. Nightly rhythms of single and multiple mounting occurred throughout the year while only two copulation events were recorded. Three months after both copulation events, the birth of porcupettes (porcupine < 5 kg) was recorded. A stochastic phase characterized by an articulate courtship with distinct behavioural patterns always preceded each mounting event. After the stochastic phase, the presenting of the female evoked by Nose-Quill contact behaviour, occurred in 83.8% (n = 182) of mounting events, while in 16.1% (n = 35) a spontaneous presenting of the female occurred. The average duration of copulation lasted 24 s (SD = 7 s) with 17 thrusting (SD = 5.5 thrusting). Births of porcupettes occurred throughout the year from 1 to 3 times *per* pair *per* year and the litter size observed ranged from 1 to 2 porcupettes. The first emerging of porcupettes from burrow occurred at 10–15 days after birth. Parents and sub-adults of the family actively perform parental care and the permanence of sub-adults within the family (i.e., from birth to dispersal) resulted to be at least 1 year. This investigation provides new useful insights on porcupine reproductive behaviour in the wild. Such new knowledge may be useful to the management of porcupines in wild, semiwild and captive condition as well as to delineate the key habitat *desiderata* of this rodent.

## Introduction

The hystricomorph rodents of genus *Hystrix*: the crested porcupine (*H. cristata*)*,* the cape porcupine (*H. africaeaustralis*) and the Indian crested porcupine (*H. indica*) show a social organization based on small family clans^1–4^ in which monogamy appears to be the mating system^1,5–8^.

The hystricomorphs are the only monogamous mammals to exhibit socio-sexual behaviour also outside the breeding period^9^. Captive crested porcupine, cape porcupine and Indian crested porcupine breed throughout the year^10–12^ and only in captive cape porcupine a peak of litters was recorded between March and August^2,11^. The reproduction period in wild crested porcupine and Indian crested porcupine is still not clear and there is a lack of data concerning birth periods in these two species. Reproduction in wild crested porcupine and Indian crested porcupine is reported not to be season dependent^13–15^. In wild Indian crested porcupine, data obtained from captured animals indicate that reproduction takes place continuously at least from April to September^16^. Conversely, free-ranging cape porcupines reproduce seasonally in summer rainfall areas with a peak of births occurring between March and August while males are reproductively active throughout the year^17–19^. For captive crested porcupine and Indian crested porcupine, copulation occurs independently from the oestrus state with nightly rhythms of mounting recorded after or before births and even in presence of porcupettes^7,10,13,14^ while copulation was observed in captive cape porcupine only during the oestrus state^3^. No data are yet available concerning mounting and copulation behaviour in all the three species of porcupine of genus *Hystrix* in wild. In captivity, in all the three species mounting and copulation behaviour are part of an elaborate courtship with free combination of a wide range of behavioural patterns: Resting, Sniffing, Grooming, Sound, Stepping, Following and Nose-Quills contact^3,7,13,14^. These behaviour patterns occur without strict rigidity in the possible combination performed and only for captive crested porcupine the mounting and copulation ethogram is available^13,14^.

Gestation in all the three porcupine species of genus *Hystrix* seems to be three months^10–12,20^. In captivity, birth occurred from two to three times/year in crested porcupine^10^, one time/year in cape porcupine^11^ and two times/year in Indian crested porcupine^12^. In wild cape porcupine and Indian crested porcupine birth of purcupettes occur up to two time/year^2,16^ while no data are available for free-ranging crested porcupine. Nevertheless, Mori et al.^15^ reported that the presence of porcupettes in burrow occurred throughout the year with a peak of presence in February and in October. Births takes place inside the burrows and the litter size ranges from one to two and occasionally three porcupettes for crested porcupine, cape porcupine and Indian crested porcupine both captive and wild^5,10,11,15,16,20^. Litters of four porcupettes were observed only in captive Indian crested porcupine^12^. In captive crested porcupine, the first emerging time of porcupettes from burrow was recorded at 10 days after birth^10^ while it occurs 40–50 days after birth in the wild^15^. No data are available concerning the time of first emerging of porcupettes from burrow for both wild and captive cape porcupine and Indian crested porcupine. In captivity, in all three species of porcupine of genus *Hystrix*, the female lactates the porcupettes for about 40–50 days and the weaning starts at ~ 3 months^10,11,20^. However, in captivity porcupettes of crested porcupine start feeding on solid food at 1-month old^10^. The length of lactation period and the beginning of weaning in the wild are not known in all the three species of porcupine. In captive crested porcupine, cape porcupine and Indian crested porcupine both parents equally perform parental care^6,10,21–23^. Male and female alternatively take care of the porcupettes (*Baby-sitting*) and frequently show allo-and mutual-grooming with the porcupettes^10,22^. Parental care was also performed by both parents in wild crested porcupine and cape porcupine^6,15^ while no data are available for the Indian crested porcupine. In captive crested porcupine, *baby-sitting* by sub-adults of the same family was also observed during porcupettes weaning phase^10^. In crested porcupine, cape porcupine and Indian crested porcupine, the time of permanence, from birth to dispersal, of sub-adults with the family seems to range from 1 to 2 years according to when they reach sexual maturity^10,12,21^.

The crested porcupine is a naturalized rodent of African origin and it is widely distributed across peninsular Italy and in the Islands of Sicily, Sardinia and Elba^24,25^ nevertheless, very little is known on its reproduction biology. So far, due to their elusive, burrowing, mainly nocturnal habits^26–29^ the available data on porcupine reproduction only apply to captive specimens^10,13,14^. The aim of this study was to investigate unknown aspects of crested porcupine in the wild and assess if wild crested porcupine reproduction behaviour differs from captive ones. This study focused on three main aspects of crested porcupine reproduction in the wild: (I) Describe frequency and mode of the mounting and copulation sequence occurring and determining whether the mounting sequence is independent from copulation ones, (II) determine the breeding period throughout the year and assess if there is a seasonality in porcupettes birth, III) Assess whether also sub-adults perform baby-sitting either alone or in collaboration with the parents.

## Results

Among individuals belonging to the eight monitored porcupine families, 12 were captured and individually marked and seven were recognisable due to the presence of phenotypic peculiarities (see Supplementary Table S1).

### Mounting and copulation behaviour

**Figure 1:**
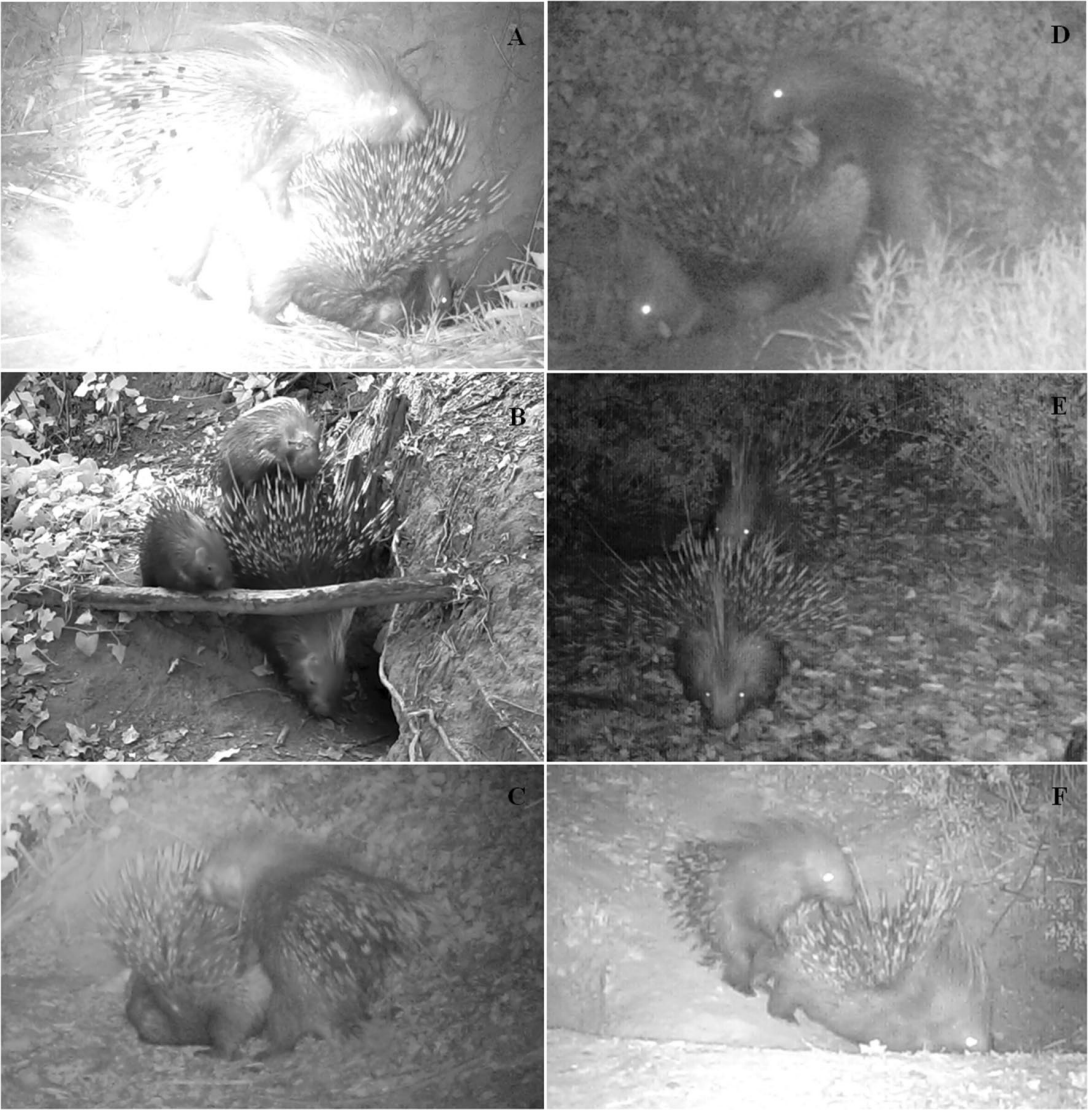
Mounting behaviour events recorded in Pair 2 (**A**), in Pair 5 (**B**), in Pair 1 (**C**) in Pair 4 (**D**), in Pair 7 (**E**) and in Pair 8 (**F**). Marked porcupines or porcupines with phenotypic peculiarities are visible.

Within 813 video recordings collected, 217 mounting and two copulation events were recorded (Fig. 1). A single mounting was recorded in 80.2% (n = 174) of events, while multiple mounting was recorded in the 19.8% (n = 43) of events (Table 1).

**Table 1:** Total number of intrapair mounting (*SM* single mounting, *MM* multiple mounting) and copulation events recorded in each monitored porcupine pair.

	Mounting events (n)	SM events (n)	MM events (n)	Copulation events (n)
Pair 1	76	56	20	0
Pair 2	21	18	3	0
Pair 3	5	5	0	0
Pair 4	12	9	3	1
Pair 5	52	41	11	1
Pair 6	8	8	0	0
Pair 7	8	8	0	0
Pair 8	35	29	6	0

**Figure 2:**
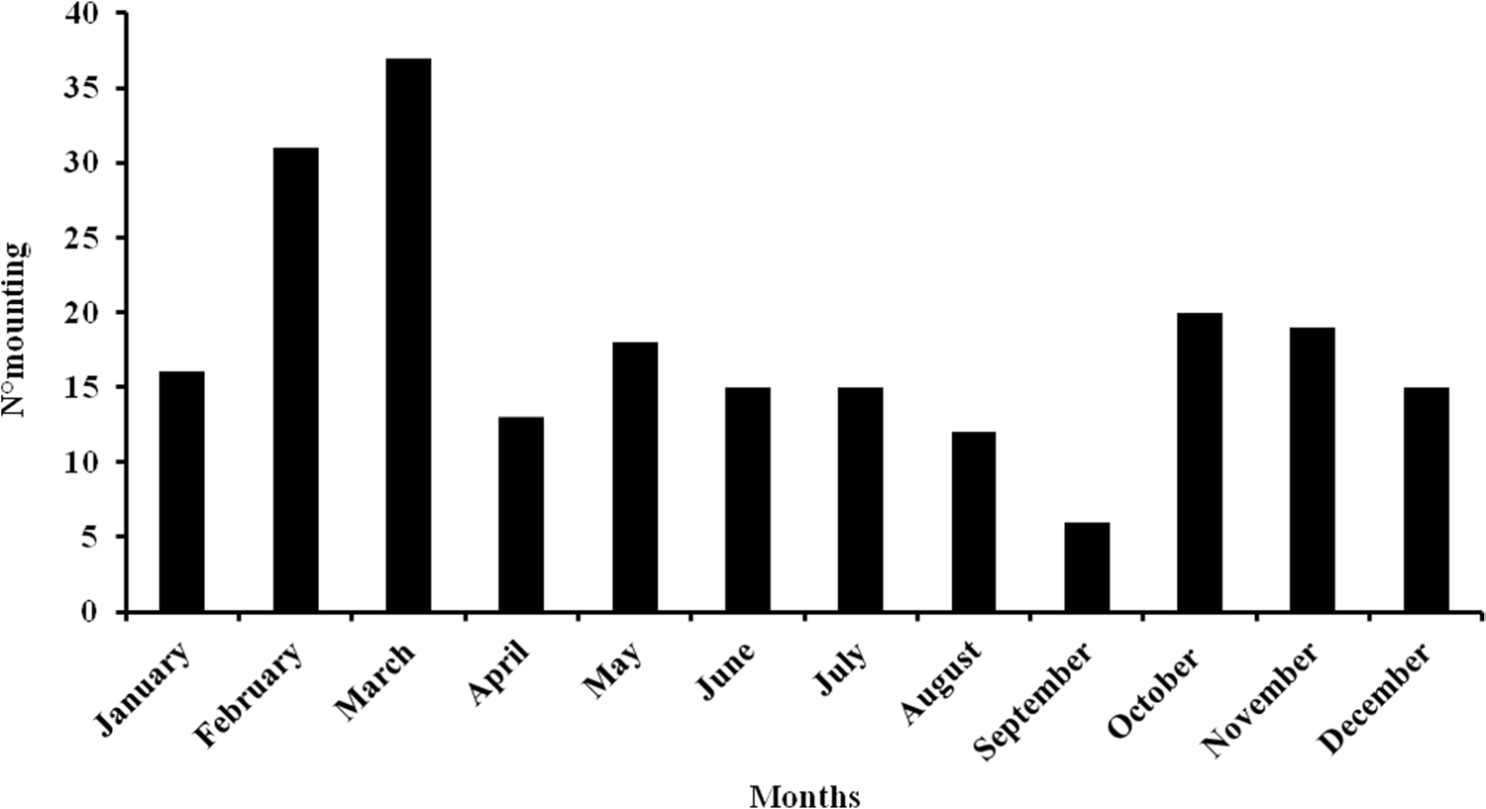
Total number of recorded mounting events *per* month for all the 8 monitored porcupine pairs during the 3 years of monitoring.

**Figure 3:**
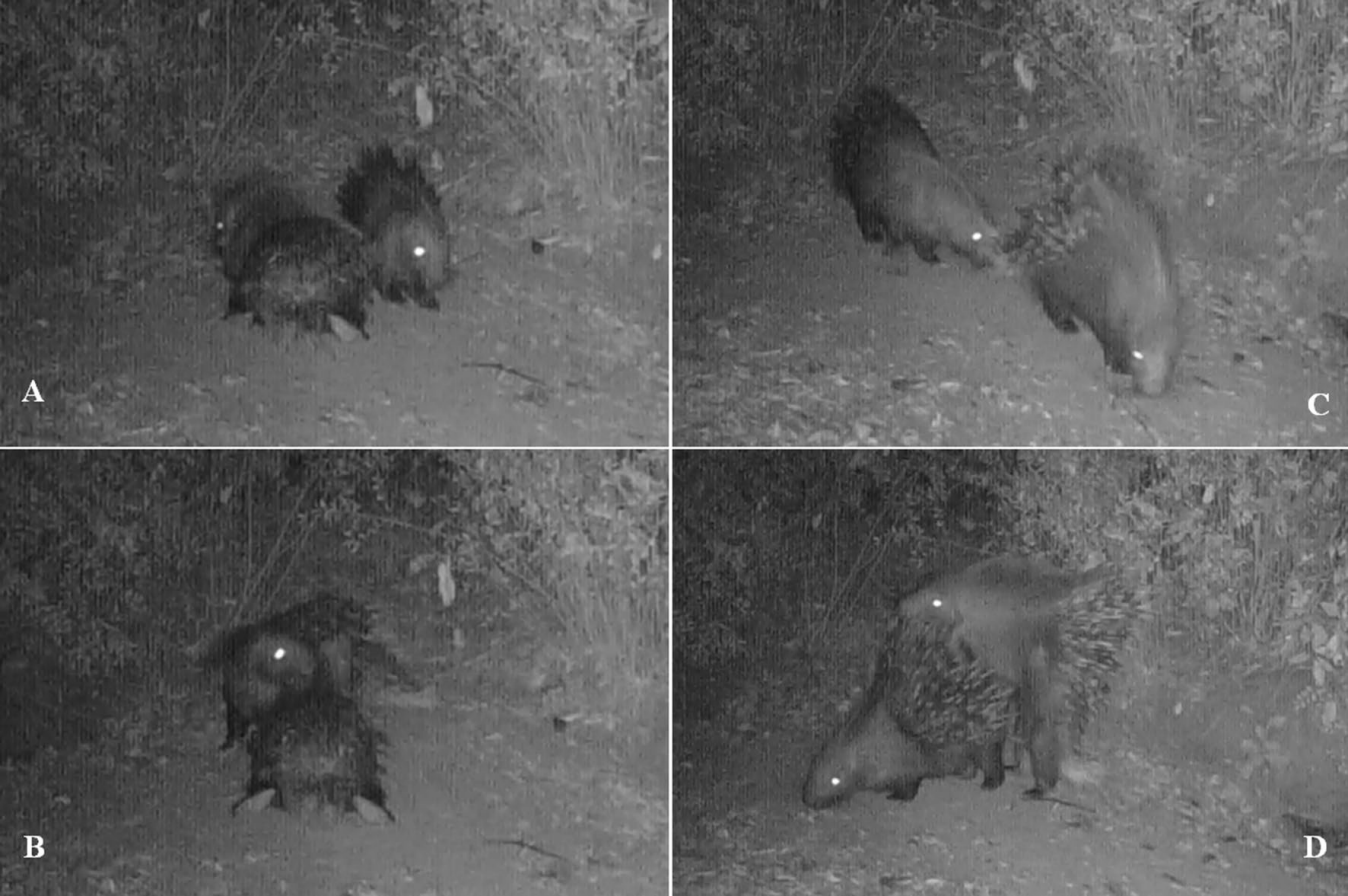
Mounting behavioural sequence observed in Pair 7: (**A**) resting behaviour, (**B**) Mutual-allo-grooming behaviour, (**C**) following associated with Nose-quills contact behaviour (**D**) and mounting event.

**Figure 4:**
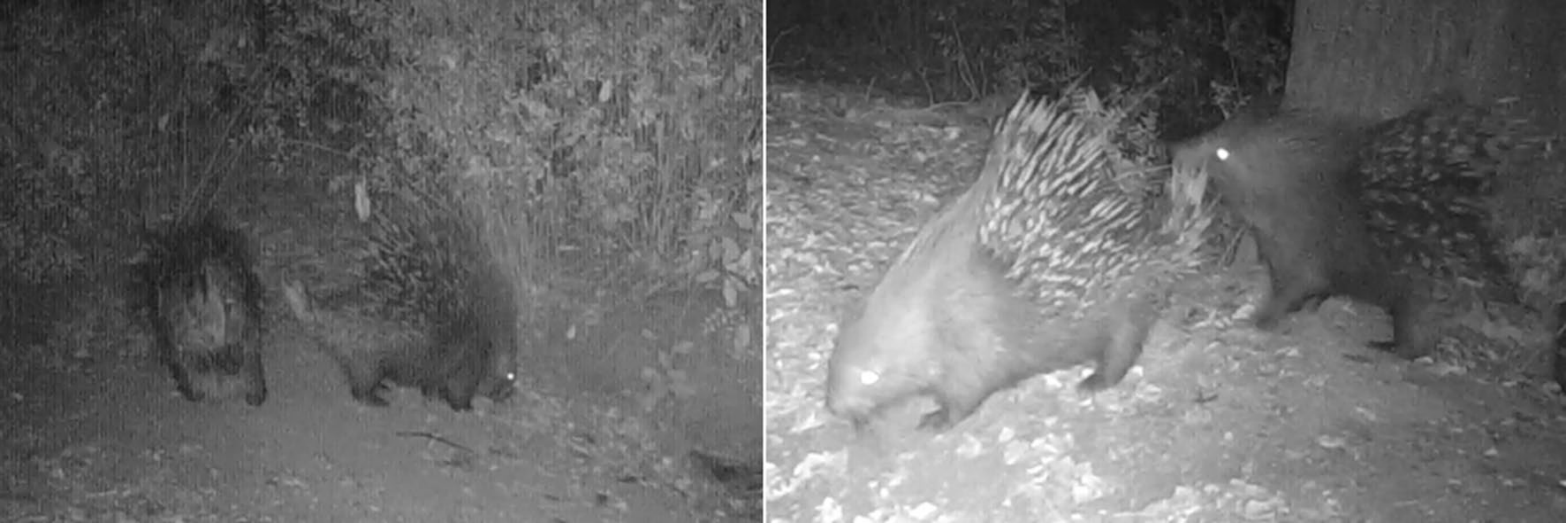
Spontaneous presenting behaviour of the female *vs* male observed in Pair 7 (left) and presenting behaviour of the female evoked by nose-quills contact behaviour in Pair 8 (right).

Mountings were recorded throughout the year even after birth, and during lactation (Fig. 2). The behavioural pattern such as Resting, Grooming, Sniffing, Sound, Stepping and Following always characterized a stochastic phase before the mounting event (Fig. 3). After the stochastic phase, the presenting behaviour of the female evoked by Nose-Quill contact was observed in 83.8% (n = 182 events) of mounting events (see Supplementary Video 1), while in 16.1% (n = 35) of events spontaneous presenting of the female without Nose-Quill contact occurred (Fig. 4). Presenting of the female, whether evoked or spontaneous, was always followed by mounting.

Presenting of the female was characterized by the back quills erection, raising of the tail towards her back and exposure of the ano-genital region. The 2 copulation events occurred in Pair 4 and 5. In Pair 4 within the copulation event three consecutive copulations were recorded in November 2018 while in Pair 5 a copulation was observed in January 2019 (see Supplementary Video 2). In Pair 4 spontaneous presenting of the female before the copulation event was observed while in Pair 5 it was not possible to assess whether copulation was evoked or not. In both pairs, 3 months after the copulation event, birth of porcupettes was recorded. The average duration of copulation was 24 s (SD = 7 s) with an average of 17 thrusting (SD = 5.5 thrusting).

### Birth

**Figure 5:**
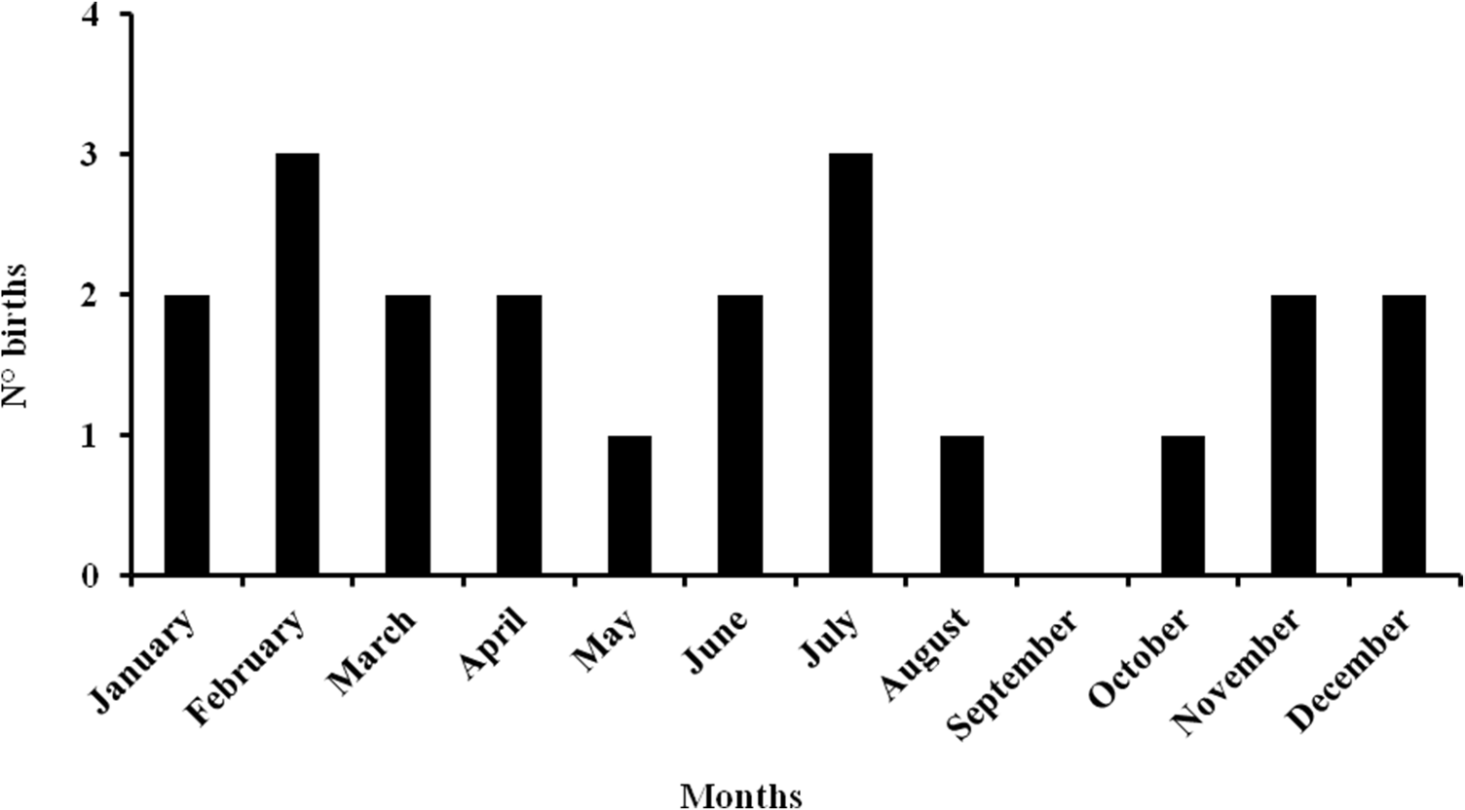
Total number of recorded births *per* month for all the 8 monitored porcupine pairs during the 3 years of monitoring.

A total of 35 porcupettes in 21 births was recorded during the study period. Births were recorded throughout the year (Fig. 5). The number of births *per* pair *per* year ranged from 1 to 3 with an average of 1.7 births (SD = 0.7 births). Seven litters consisted of only one porcupette and 14 of two porcupettes, with a recorded average of litter size of 1.6 porcupettes (SD = 0.5 porcupettes). Litters exceeding 2 porcupettes have never been recorded.

The estimated age of porcupettes at first emergence from the burrow ranged from 10 to 15 days after birth. During 10 days prior to porcupettes emerging, the male and the female alternately emerged from burrow during the night hours and increased the frequency of burrowing visiting. Meanwhile little activity of the female outside the burrow was recorded in all the monitored pairs. In 6 occasions, the adult male brought food (n = 4) or bones (n = 2) into the burrow, just before the first emerging of porcupettes.

**Figure 6:**
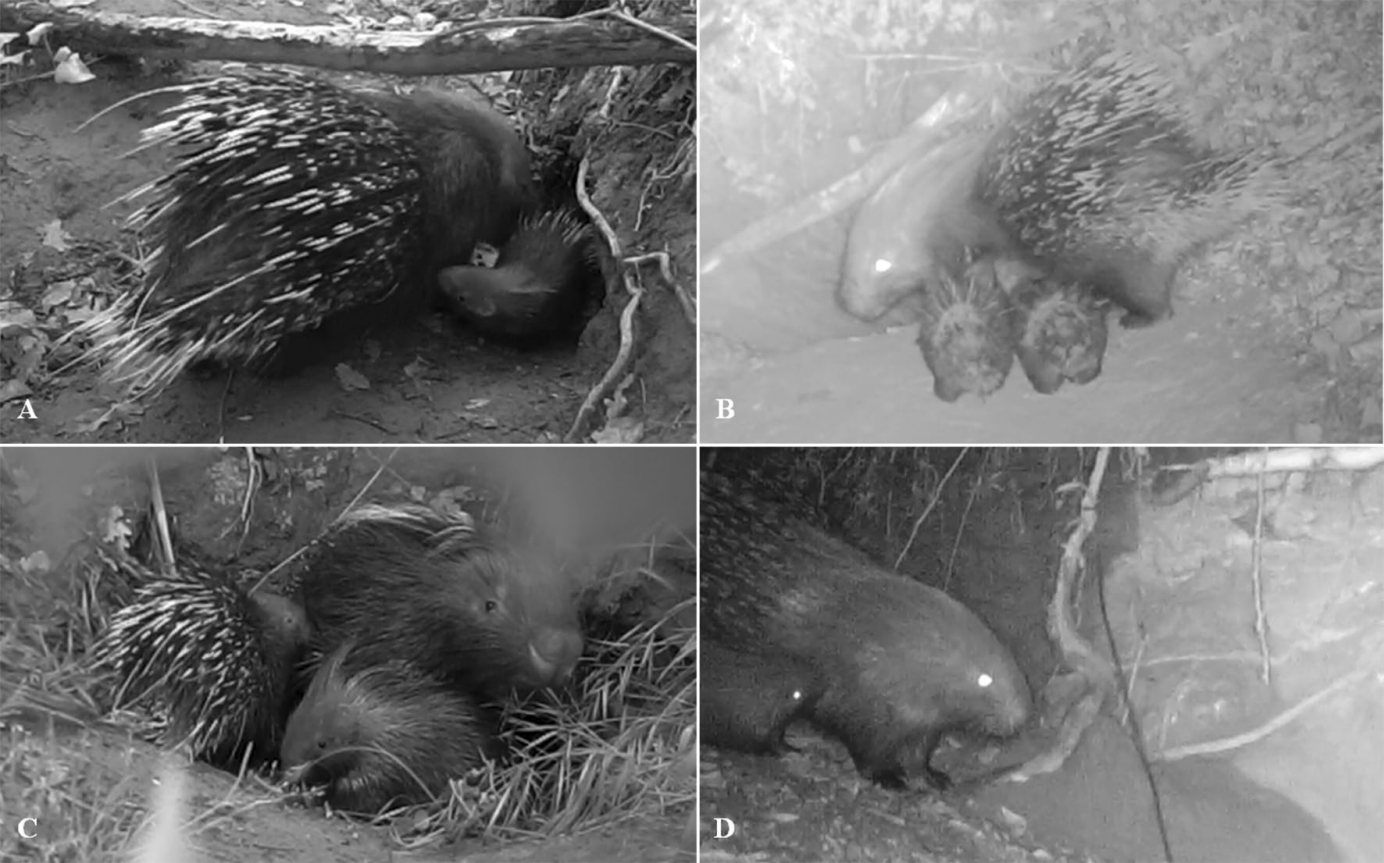
Adult female of Pair 4 (**A**), of Pair 1 (**B**), of Pair 2 (**C**) and of Pair 3 (**D**) while nursing porcupettes.

The porcupettes were observed to be eating independently after 10–15 days from first emerging nevertheless nursing events were recorded up to 2 months after the first emerging (Fig. 6).

### Parental care

A total of 2039 events of parental care by the adult male, the adult female, sub-adults and adult male and female together, were recorded from the monitored porcupine families (Table 2). Baby-sitting by adult male was observed since first emergence of porcupettes. Sub-adults also actively took part in the care of the litter in absence of parents. Parental care by sub-adults, the adult male and female together were recorded in all the monitored porcupine families. The frequency of parental care by the adult male and the adult female did not show statistical differences in all monitored porcupine pairs (Family 1: χ^2^ = 0.024, *p* = 0.876853; Family 2: χ^2^ = 0.073, *p* = 0.787082; Family 3: χ^2^ = 0.4372, *p* = 0.508462; Family 4: χ^2^ = 0.3104, *p* = 0.577436; Family 5: χ^2^ = 0.3963, *p* = 0.529003; Family 8: χ^2^ = 0.308, *p* = 0.578897). The minimum time of permanence of the five recognisable sub-adults within the family was about 1 year.

**Table 2:** Total number of parental care events recorded in each monitored porcupine family.

	Events	M (%)	F (%)	M + F (%)	SA (%)	ND (%)
Family 1	576	17.4 (n = 100)	17.7 (n = 102)	59.5 (n = 343)	0.5 (n = 3)	4.9 (n = 28)
Family 2	433	16.9 (n = 73)	17.5 (n = 76)	24.5 (n = 106)	27.5 (n = 119)	13.6 (n = 59)
Family 3	548	15.2 (n = 83)	16.6 (n = 91)	28.8 (n = 158)	17.9 (n = 98)	21.5 (n = 118)
Family 4	102	15.7 (n = 16)	18.6 (n = 19)	30.4 (n = 31)	6.9 (n = 7)	28.4 (n = 29)
Family 5	236	17 (n = 40)	14.8 (n = 35)	38.6 (n = 91)	16.5 (n = 39)	13.1 (n = 31)
Family 8	144	10.5 (n = 15)	12.5 (n = 18)	59.0 (n = 85)	9.0 (n = 13)	9.0 (n = 13)

The frequency of parental care occurrence by the adult male (M), the adult female (F), sub-adults (SA), and the adult male and female together (M + F) in each family are reported. Numbers of parental care events performed by undetermined individuals (ND) are also reported.

## Discussion

Outdoor mounting events in free-ranging crested porcupines occur during night hours throughout the year independently from birth, lactation and presence of porcupettes. Nightly rhythms of single mounting are regularly performed and multiple mountings *per* night were frequently observed. These results are in accordance with what observed in captive crested porcupine^13,14^ and in captive Indian crested porcupine^7^. This suggests that mounting behaviour in crested porcupine is not linked to the oestrus state and is probably performed as a mechanism for pair maintaining as suggested by Weir^5,13^. The sequence of mounting and the behavioural displays recorded in free-ranging crested porcupines was the same as described in captivity by Felicioli et al.^13,14^. The observations of this study confirm that the presenting of the female for mounting is usually induced by the Nose-quill contact by the male.

Copulation time in wild porcupine recorded in the present study, was longer than in captive porcupine^13^. Longer copulation time (2–3 min) was observed in captive cape porcupine^3^. The spontaneous presenting of the female could be a signal of oestrus status as hypothesized by Kleiman^9^ and by Felicioli et al.^13^ in captive crested porcupine. Time between copulation and porcupettes birth allowed us to hypothesise that in crested porcupine copulation occurs during the oestrus state. However, since only two events of copulation were recorded in this investigation it is still not possible to exclude that copulation could occur also outside the oestrus period as hypothesized by Felicioli et al.^13^ in captive crested porcupine and by Sever and Mendelsshon^7^ in captive Indian crested porcupine.

Births of porcupettes was observed throughout the year as previously reported by Santini^20,30^ in free-ranging porcupines and by Grazzini^10^ in captive ones. Observations performed in this study indicate the absence of a seasonal effect in crested porcupine birth period. The number of births 1 to 3 *per* year recorded in the current study is in accordance with the results obtained by Grazzini^10^ in captive crested porcupine. The litter size of 1 to 2 porcupettes *per* birth as recorded in our study was also reported in other studies^5,10,11,15,16,20^, however, occasional litters of three porcupettes in free ranging crested porcupines were reported by Santini (unpublished data) and Mori et al.^15^ and of four porcupettes in captive Indian crested porcupine^12^.

The first emergence age range recorded in this investigation in free-ranging crested porcupine clearly differs from that reported by Mori et al.^15^ (about 40–50 days), while it results similar to that observed in captive crested porcupine^10^. This can be attributed to the different observation pattern, as Mori et al.^15^ visited a burrow once in a month, while our observation was based on a continuous monitoring using camera-traps. The porcupettes age range of first emergence estimated in this study is further supported by the observation of porcupettes eating solid food 30 days after birth. The higher frequency of burrow visiting by the adult male and female just before first emerging of porcupettes was also observed in captive^31^ and free-ranging^15^ crested porcupine. At the same time, the reduced activity of the female outside the burrow during this period, while the adult male brings food into the burrow, allows us to hypothesise that females mostly remain inside the burrow to give birth while the male looks after its partner. No significant differences arise between the male and the female baby-sitting, confirming that both parents are equally involved in parental care as also reported by Grazzini^10^ in captive crested porcupine and Mori et al.^15^ in wild crested porcupine. Baby-sitting by sub-adults was also previously observed in captive crested porcupines by Grazzini^10^. Permanence of sub-adults within the family until one year old observed in this study confirms what hypothesized by Grazzini^10^ in captive crested porcupine and in captive cape porcupine by Van Aarde^21^. The age of maturity for crested porcupine is 9–10 months^20^, while it is one year old for cape porcupine^21^. Departure of sub-adults from family is linked to physiological needs. However, no information is available concerning the dispersal of porcupine’s sub-adults.

In conclusion the results obtained in this investigation indicate that the mounting and copulation are two distinct behaviours in wild crested porcupine. Mounting events occur in the night throughout the year as a ritual that allows pair bond maintenance while further investigation is required to assess if copulation is linked to oestrus state. The birth of porcupettes throughout the year suggests that oestrus in crested porcupines is not season-dependent and parents and sub-adults of the same family actively perform baby-sitting together as well as alone.

## Materials and methods

### Study area and camera-trapping

The investigation was performed between 2017 and 2019 in a hilly area of 2548 ha in Crespina-Lorenzana (43.57181 Lat., − 10.55348 Long.) in the province of Pisa (Tuscany, Central Italy). Such area is characterized by high biodiversity and environmental fragmentation in which small woody areas are interspersed with uncultivated and/or cultivated areas and rivers. The camera-trapping monitoring was performed in eight settlements, each inhabited by a recognisable porcupine family ranging between four and seven individuals. Six porcupine capture campaigns were performed between 2017 and 2018 in order to individually mark the porcupines inhabiting the monitored settlements in order to make them recognisable in the videos recorded by camera-traps. Porcupines capture and handling were carried out in accordance with the guidelines and protocol approved by the Italian Institute for Environmental Protection and Research (ISPRA) with protocol number 22584 of 8th May 2017 and by Tuscany Region with the Decree n. 14235 of 3rd October 2017.

The porcupines were trapped in wire mesh cages with double entrance (110 × 42 × 42 cm) and baited with corn and potatoes as described by Coppola et al.^32^. Each captured porcupine was anaesthetized using a 4–6 mg/kg of tiletamine-zolazepam (Zoletil) in accordance with the protocol of Coppola et al.^33^, weighted, sexed and individually marked by coloured adhesive tapes applied on the quills, by white or black non-toxic water-based paint sprayed on the crest and/or on the tail or else by a combination of these. Coloured adhesive tapes didn’t need to be removed but were gradually lost by the animals through the natural process of dropping of the quills. The marking with coloured adhesive tape remains clearly visible for at least 1 year while the paint lasted about 20 days in absence of rain. The age class of each captured porcupine was estimated on the base of animal weight^12^. Moreover, the individual recognition of some unmarked specimens was possible due to the presence of phenotypic peculiarities such as blindness, scars and injuries. Cameras-traps (Num’axes PIE1009) with passive infrared sensor (PIR) were continuously and simultaneously activated in all monitored settlements, for a total of 913 trap days. Each settlement was sampled by using one camera-trap deployed at a minimum distance of 1.5–2.5 m at a height of 1.5 m from burrow entrance holes where clear signs of activity were present. Each camera-trap was set up to record 20 s videos without time lapse and to stamp date and hour in each video recorded. Camera-traps videos recordings were checked and filed on a weekly basis and analysed by an expert.

### Reproductive behaviour

#### Intrapair mounting and copulation behaviour

For each monitored porcupine family only those videos recording the adult pair performing mounting and/or copulation were used to describe these behaviours in free-ranging porcupines. The mounting and copulation sequences were observed and described following the mounting and copulation sequence ethogram reported by Felicioli et al.^13^ for the same species in captivity. The mounting was defined when male lifted his body in an upright position extroflecting the penis and bipedally advancing towards the female until the underside part of her raised tail makes support and contact with male belly, the forelegs do not hold the female’s back^3^. Copulation was defined as insertion of the penis into the vagina. In accordance with Felicioli et al.^13^ the mounting behaviour was assessed when intromission (insertion of the penis into the vagina) and thrusting (pelvic movements during intromission) doesn’t occur. Conversely, the copulation was assessed if intromission and thrusting was observed. For each monitored reproductive pair the number of total, single and multiple mounting events as well as copulation events observed were recorded. Consecutive videos clearly attributable to the same mounting or copulation of the same porcupine pair were considered as a single event. At the same time repeated mounting or copulation in single or consecutive video recordings were considered as a single event. Whenever possible, the duration of copulation and number of thrusting were also recorded. The sequences of behavioural patterns (i.e., Resting, Sniffing, Grooming, Sound, Stepping, Following and Nose-Quills contact) performed during each mounting event observed and the frequency of occurrence of the different sequences were recorded and described.

### Birth

Birth data were collected from all the eight recognisable porcupine pairs. For each monitored reproductive pair the number of births occurred throughout the whole period of monitoring was recorded. For each birth event, the month and the litter size were recorded. In addition, the age of porcupettes at the first emergence from burrow after birth were estimated based on the time of appearance of peculiar behaviours in the adults (i.e., increase in the frequency of burrow visiting by the adults during the night, bringing food into the burrow). Whenever possible the time interval between the first emerging of porcupettes from the burrow and the first time in which they were observed eating independently was recorded.

### Parental care

Data concerning the parental care (i.e., time spent with porcupettes) were collected from six out of eight recognisable porcupine families. The parental care was assessed by using the camera-traps videos in which porcupettes with adults or sub-adults of the family were simultaneously present. Consecutive videos clearly attributable to the same porcupine individuals were considered as a parental care event. Total number of parental care events observed and the frequency of parental care occurrence involving the adult male, the adult female, sub-adults and adult male and female together were recorded in each investigated family. The difference of occurrence of parental care by the male and the female in each family was analysed using chi-square test (χ^2^) in JMP software (SAS Institute, Cary, NC, USA 2008). The minimum time of permanence of marked and/or recognisable sub-adults within the family was also detected. The minimum permanence was assessed considering the last time in which marked sub-adults were detected on camera traps videos with the family.

### Ethics approval

The capture-marking activity of porcupines and animal handling protocol were approved by the ethics committee of Italian Institute for Environmental Protection and Research (ISPRA) with protocol number 22584 of the 8 May 2017 and by Tuscany Region with the Decree n. 14235 of the 3 October 2017. This study was carried out in compliance with the ARRIVE guidelines.

## Supplementary Information

Supplementary Table S1.

Supplementary Legends.

Supplementary Video 1.

Supplementary Video 2.

## Data Availability

All data are available on request to the corresponding author.

## References

[1] Mohr E (1965). Altweltliche Stachelschweine.

[2] Kingdon J, Kingdon J (1974). Porcupines (*Hystrix*). East African Mammals.

[3] Morris DJ, Van Aarde RJ (1985). Sexual behavior of the female porcupine *Hystrix africaeaustralis*. Horm. Behav..

[4] Saltz D, Alkon PU (1992). Observations on den shifting in Indian crested porcupine in the Negev (Israel). Mammalia.

[5] Weir BJ, Rowlands IW, Weir BJ (1974). Reproductive characteristics of Hystricomorph rodents. The Biology of Hystricomorph Rodents. Symposia of the Zoological Society of London.

[6] van Aarde RJ (1987). Reproduction in the Cape porcupine *Hystrix africaeaustralis:* An ecological perspective. S. Afr. J. Sci..

[7] Sever Z, Mendelssohn H (1988). Copulation as a possible mechanism to maintain monogamy in porcupines, *Hystrix indica*. Anim. Behav..

[8] Sever, Z. & Mendelssohn, H. Nightly copulations throughout the year in monogamous porcupines. In *2nd International Conference on Behavioral Ecology, Vancouver, Canada* (1988)

[9] Kleiman DG, Rowlands IW, Weir BJ (1974). Patterns of behaviour in Hystricomorph rodents. The Biology of Hystricomorph Rodents. Symposia of the Zoological Society of London.

[10] Grazzini, M.T. Comportamento riproduttivo e accrescimento post-natale in *Hystrix cristata* L. (Rodentia, Hystricidae). Dissertation, University of Pisa (1992).

[11] van Aarde RJ (1985). Reproduction in captive female Cape porcupines *(Hystrix africaeaustralis)*. J. Reprod. Infertil..

[12] Tohmè G, Tohmè H (1980). Contribution à l’ètude du porc-èpic *Hystrix indica indica* Kerr, 1972 (Rodentia). Mammalia.

[13] Felicioli A, Grazzini A, Santini L (1997). The mounting and copulation behaviour of the crested porcupine *Hystrix cristata*. Ital. J. Zool..

[14] Felicioli A, Grazzini A, Santini L (1997). The mounting behaviour of a pair of crested porcupine *H. cristata* L.. Mammalia.

[15] Mori E, Menchetti M, Lucherini M, Sforzi A, Lovari S (2016). Timing of reproduction and paternal cares in the crested porcupine. Mamm. Biol..

[16] Kadhim AHH (1997). Distribution and reproduction of the Indian Crested Porcupine *Hystrix indica* (Hystricidae: Rodentia) in Iraq. Zool. Middle East.

[17] Skinner JD, van Aarde RJ, van Jaarsveld AS (1984). Adaptations in three species of large mammals (*Antidorcas marsupialis*, *Hystrix africaeaustralis*, *Hyaena brunnea*) to arid environments. S. Afr. J. Zool..

[18] Van Aarde RJ, Skinner JD (1986). Reproductive biology of the male Cape porcupine, *Hystrix africaeaustralis*. J. Reprod. Fertil..

[19] van Aarde, R.J. Reproduction in the porcupine *Hystrix africaeaustralis* Peters. Dissertation, University of Pretoria (1984).

[20] Santini L (1980). The habits and influence on the environment of the old world porcupine *Hystrix cristata* L. in the northernmost part of its range. Proc. 9th Vertebr. Pest Conf..

[21] van Aarde RJ (1987). Pre- and post natal growth of the Cape porcupine *Hystrix africaeaustralis*. J. Zool. Lond..

[22] Sever Z, Mendelssohn H (1989). Paternal behavior in porcupines. Isr. J. Zool..

[23] Sever Z, Mendelssohn H (1991). Time Budget of Paternal Behaviour in Monogamous Porcupines.

[24] Vecchio G, Coppola F, Scarselli D, Giannini F, Felicioli A (2018). Crested porcupine in the Island of Elba, Italy: Native or alien?. Curr. Sci..

[25] Trucchi E, Facon B, Gratton P, Mori E, Stenseth NC, Jentoft S (2016). Long live the alien: Is high genetic diversity a pivotal aspect of crested porcupine (*Hystrix cristata*) long-lasting and successful invasion?. Mol. Ecol..

[26] Pigozzi G (1986). Crested porcupines (*Hystrix cristata*) within badger setts (*Meles meles*) in the Maremma Natural Park, Italy. Saugetierk. Mitt..

[27] Coppola F, Vecchio G, Felicioli A (2019). Diurnal motor activity and “sunbathing” in crested porcupine (*Hystrix cristata* L., 1758). Sci. Rep..

[28] Coppola F, Dari C, Vecchio G, Scarselli D, Felicioli A (2020). Cohabitation of settlements among crested porcupine (*Hystrix cristata*), red fox (*Vulpes vulpes*) and European badger (*Meles meles*). Curr. Sci..

[29] Coppola F, Guerrieri D, Simoncini A, Varuzza P, Vecchio G, Felicioli A (2020). Evidence of scavenging behaviour in crested porcupine. Sci. Rep..

[30] Santini L (1983). I Roditori Italiani di Interesse Agrario e Forestale.

[31] Felicioli, A. Analisi spazio-temporale dell’attività motoria in *Hystrix cristata* L. Dissertation, University of Pisa (1991).

[32] Coppola F, D’Addio E, Casini L, Sagona S, Aloisi M, Felicioli A (2020). Hematological and serum biochemistry values in free-ranging crested porcupine. Vet. Sci..

[33] Coppola F, D’Addio E, Casini L, Sagona S, Felicioli A (2020). Field chemical immobilization of free-ranging crested porcupines with Zoletil®: A reviewed dosage. Vet. Sci..

