# Supplementary material for: Reproductive behaviour in free-ranging crested porcupine Hystrix cristata L., 1758
Source: Sci Rep. 2021 Oct 11;11:20142. doi: 10.1038/s41598-021-99819-3 (PMC8505399; doi:10.1038/s41598-021-99819-3)
Supplement: Supplementary file 1 — Supplementary Table S1. [file 41598_2021_99819_MOESM1_ESM.pdf]

Supplementary Table S1. Porcupines individually marked and/or recognisable for the presence of phenotypic peculiarities in each porcupine family group. For the individuals phenotypically recognisable the phenotypic characteristic is reported.

	Specimens	Marked	Recognisable
**Family 1**	Adult male	X	White tapes on the quills<br>Blindness left and right
	Sub-adult female	X	Red tapes on the quills<br>Black paint on the crest
	Porcupette male	X	White paint on the tail
**Family 2**	Adult male	X	Black tapes on the quills<br>Blindness left
	Adult female	NO	Blindness left
	Porcupette female	X	White tapes on the quills
	Sub-adult	NO	Absence of crest
**Family 3**	Adult female	NO	Blindness left
	Adult male	NO	Blindness right
**Family 4**	Adult male	X	Black tapes on the quills<br>Black paint on the tail
	Sub-adult female	X	Red tapes on the quills<br>White paint on the tail
**Family 5**	Adult male	X	White tapes on the quills<br>Injury on the nose
	Adult female	NO	Crest carried on the left side
**Family 6**	Adult female	X	Black tapes on the quills
	Adult male	NO	Injury in the rump (left)<br>Blindness right
**Family 7**	Adult female	X	Red tapes on the quills<br>White paint on the crest
**Family 8**	Sub-adult female	X	White tapes on the quills
	Porcupette female	X	Black tapes on the quills
	Sub-adult male	NO	Injuries in the rump (left)
