# Supplementary material for: Reproductive behaviour in free-ranging crested porcupine Hystrix cristata L., 1758
Source: Sci Rep. 2021 Oct 11;11:20142. doi: 10.1038/s41598-021-99819-3 (PMC8505399; doi:10.1038/s41598-021-99819-3)
Supplement: Supplementary file 2 — Supplementary Legends. [file 41598_2021_99819_MOESM2_ESM.docx]

**Supplementary video captions**

**Supplementary Video S1:** Mounting event recorded in Pair 5 in which presenting of the female evoked by nose-quill contact behaviour is visible

**Supplementary Video S2:** Copulation and thrusting recorded in Pair 4 with spontaneous presenting of the female.
